# Phosphorylation of MSI-1 is implicated in the regulation of associative memory in *Caenorhabditis elegans*

**DOI:** 10.1371/journal.pgen.1010420

**Published:** 2022-10-12

**Authors:** Pavlina Mastrandreas, Csaba Boglari, Andreas Arnold, Fabian Peter, Dominique J.-F. de Quervain, Andreas Papassotiropoulos, Attila Stetak

**Affiliations:** 1 Transfaculty Research Platform Molecular and Cognitive Neurosciences, University of Basel, Basel, Switzerland; 2 Division of Molecular Neuroscience, Department of Psychology, University of Basel, Basel, Switzerland; 3 Biozentrum, Life Sciences Training Facility, University of Basel, Basel, Switzerland; 4 Division of Cognitive Neuroscience, Department of Psychology, University of Basel, Basel, Switzerland; 5 University Psychiatric Clinics, University of Basel, Basel, Switzerland; Brown University, UNITED STATES

## Abstract

The Musashi family of RNA-binding proteins controls several biological processes including stem cell maintenance, cell division and neural function. Previously, we demonstrated that the *C*. *elegans* Musashi ortholog, *msi-1*, regulates forgetting via translational repression of the Arp2/3 actin-branching complex. However, the mechanisms controlling MSI-1 activity during the regulation of forgetting are currently unknown. Here we investigated the effects of protein phosphorylation on MSI-1 activity. We showed that MSI-1 function is likely controlled by alterations of its activity rather than its expression levels. Furthermore, we found that MSI-1 is phosphorylated and using mass spectrometry we identified MSI-1 phosphorylation at three residues (T18, S19 and S34). CRISPR-based manipulations of MSI-1 phosphorylation sites revealed that phosphorylation is necessary for MSI-1 function in both short- and long-term aversive olfactory associative memory. Thus, our study provides insight into the mechanisms regulating memory-related MSI-1 activity and may facilitate the development of novel therapeutic approaches.

## Author summary

Understanding neural circuits and molecular mechanisms underlying learning and memory are the major challenges of neuroscience. It is a generally accepted model that a learning event causes modification of synapses; strengthening some within a circuit and weakening others (termed “synaptic plasticity”). A plastic nervous system requires not only the ability to acquire and store but also to forget new inputs. While learning and memory is widely investigated, clear-cut evidence for mechanisms involved in forgetting is still sparse. Previously, we demonstrated the role of the protein Musashi (MSI-1) in the active regulation of forgetting in the nematode *C*. *elegans*. Here we investigated the role of protein modification (phosphorylation) as a possible regulatory mechanism of the MSI-1 protein activity. We found that MSI-1 protein is modified at different positions and all of these modifications at the protein level contribute to the correct activity of the protein leading to active forgetting of short and long-term memories.

## Introduction

Characterization of molecules and mechanisms underlying learning and memory is crucial for better understanding how cognitive processes are regulated. Furthermore, studies investigating the molecular mechanisms of memory acquisition, consolidation and retrieval may provide new therapeutically relevant directions to treat memory-related disorders [1–3].

In order for a plastic neural system to function properly it requires not only the acquisition but also the elimination of memories [4]. Accumulating evidence supports the existence of several parallel-acting intrinsic forgetting pathways that regulate various stages of memory processing. For instance, in the *Drosophila* mushroom body activation of Rac1 and Cdc42, members of the RhoGTPase family, increases forgetting of differentially consolidated olfactory memories via two different actin polymerization pathways; the Rac1/SCAR/Dia and Cdc42/WASp/Arp2/3 complexes [5]. On the other hand, the Drosophila-specific dopamine receptor (Damb) mediates forgetting via Gαq activation and subsequent Ca^2+^ signaling mobilization. During memory acquisition a strong dopamine release is triggered, driving both cAMP and Ca^2+^ signaling through the dDA1 and Damb receptors, whilst during forgetting a weaker DA release takes place and acts through Damb/Gαq/Ca^2+^ signaling [6]. Additionally, RhoGTPases are involved in memory decay during object recognition in mice, with elevated Rac1 activity accelerating the decay of LTP and inhibition of Rac1 prolonging it [7]. Moreover, hippocampal dopamine signaling appears to diminish the late-phase consolidation of cocaine-associated memories in rodents, thus affecting long-term memory storage [8]. Furthermore, mouse Synaptotagmin-3 is responsible for AMPA-type glutamate receptor removal from the synaptic membrane, which induces long-term depression and as a consequence, elimination of spatial memories [9]. In *C*. *elegans*, TIR-1/JNK-1 pathway mutants display accelerated forgetting of olfactory and gustatory memories mediated by changes in signal secretion and synaptic transmission between sensory neurons [10]. We previously showed that the *C*. *elegans musashi* gene ortholog, *msi-1*, actively promotes forgetting via the translational repression of members of the Arp2/3 actin branching complex, thus altering the actin cytoskeleton structure and synapse size [11]. Additionally, we showed that MSI-1 function is necessary in the AVA interneuron and lies downstream of an AMPA-type glutamate receptor (GLR-1), hence identifying a novel GLR-1/MSI-1/Arp2/3 pathway involved in memory decay [11]. Altogether, these results highlight the presence of conserved complexes and controlled mechanisms that regulate forgetting.

The *musashi* gene was originally identified as a regulator of asymmetric cell division in *Drosophila* [12]. Musashi family members are RNA-binding proteins that interact with the 3’UTR region of target RNAs in a sequence-specific manner [13]. The Musashi structure consists of two tandem positioned RNA-recognition motifs (RRMs) located at the N-terminal region of the molecule and a putative disordered region at the C-terminal end [13, 14]. Both the domain structure as well as the amino acid sequence of the RRMs are found to be evolutionarily highly conserved throughout the Musashi family members of different species. In vertebrates, the two Musashi homologues, MSI1 and MSI2, are broadly expressed in various tissues including the developing and adult nervous system [14]. Several studies suggest that Musashi proteins contribute to embryonic development and maintenance of stem cell properties by regulating components of multiple signaling pathways, including WNT [15], NOTCH [16], and TGFβ [17]. In order to fulfil these diverse cellular functions, the expression levels of MSI must be tightly controlled [18, 19]. Accordingly, several studies have aimed to identify key regulators of Musashi expression, for example, in human HPSCs [20] and during mammalian spermatogenesis [21].

Besides the regulation of MSI activity at the transcriptional and translational levels, post-translational modulation could also have a key role in the maintenance of protein homeostasis and activity. Several studies have investigated the function of post-translational modifications of Musashi orthologs. For instance, human MSI2 protein is ubiquitinated in breast cancer cells, resulting in its proteasomal degradation [22]. Other studies investigated the role of phosphorylation of MSI1 and MSI2 in *Xenopus* oocytes and showed that phosphorylation is required for the protein’s function in cell-cycle regulation [23,24]. MSI2 undergoes progesterone-dependent phosphorylation during the maturation of *Xenopus* oocytes, which is necessary for its function in translational control. Furthermore, an alternatively spliced MSI2 isoform that lacks the exon containing the phosphorylated residues fails to regulate translation of target mRNAs, suggesting that phosphorylation likely plays an important role in MSI2 function [25]. These findings highlight the importance of MSI2 phosphorylation and raise the possibility that Musashi activity may be regulated via phosphorylation in most species. Despite the apparent importance of phosphorylation that may alter the affinity of MSI-1 to other interacting proteins and/or to downstream target RNAs, or change MSI-1 protein levels through translational auto-regulation [26], its potential role during associative memory has yet to be investigated. This together with our previous findings showing that MSI-1 is highly expressed in the nervous system and is implicated in the active regulation of forgetting [11], a neuron-dependent process, prompted us to investigate the role of MSI-1 phosphorylation in olfactory associative memory.

In this study, we show that the function of MSI-1 protein in the regulation of memory is likely modulated by alterations in its activity rather than its abundance. Using mass spectrometry, we identified MSI-1 phosphorylation at amino acid residues T18, S19 and S34. To study the role of MSI-1 phosphorylation during memory, we converted the identified sites alone or in combination to alanine, thereby, preventing phosphorylation, and tested the mutant worms for short- (STAM) and long-term (LTAM) associative memory performance. Our findings indicate that both the single and the simultaneous T/S to A mutations inhibit forgetting to a similar extent as observed for the *msi-1* deletion mutant, suggesting that the phosphorylation is essential for MSI-1 function. The phospho-mimicking mutation, *msi-1(S34D)*, on the other hand, does not interfere with memory. Interestingly, *msi-1(T18D)* and *msi-1(S19D)* mutations impaired short-term but not long-term memory retention. This suggests that emulating constitutive phosphorylation at specific residues may also impair MSI-1 function during short-term memory. Altogether, we identified specific residues at the N-terminal end of *C*. *elegans* MSI-1 proteins that are phosphorylated and demonstrated that phosphorylation at these sites is necessary for the protein’s activity during memory formation.

## Results

### Total MSI-1 abundance does not change upon learning and memory consolidation

**Fig 1:**
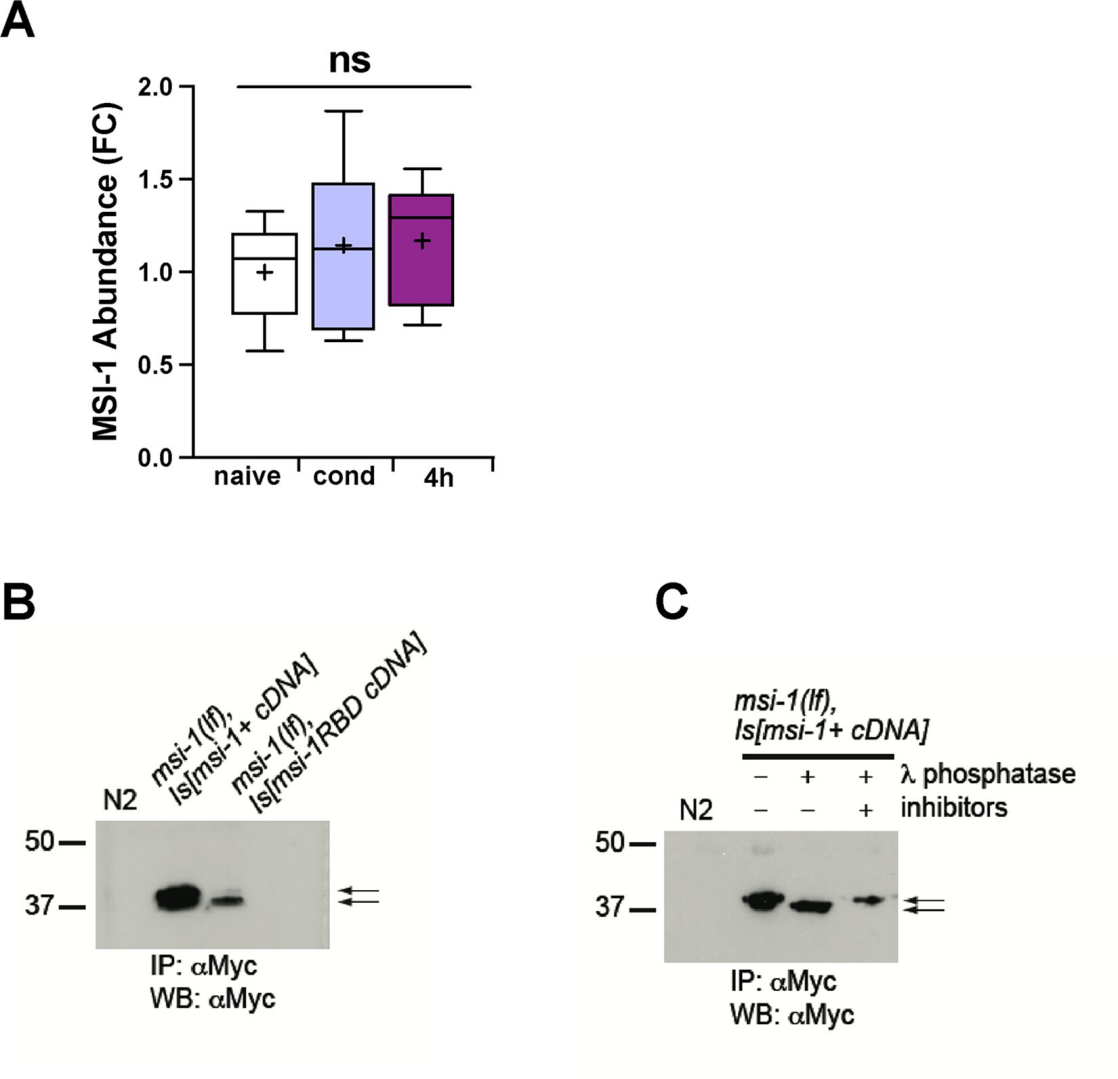
*elegans* MSI-1 is a phosphoprotein whose abundance remains constant upon learning and memory. ***C*.** (A)Worms were collected before (naive), right after (cond) or 4 hours after (4h) conditioning; MSI-1 was immunoprecipitated and analyzed with MS. Abundance of MSI-1 was estimated from calculating the summed abundance of all detected MSI-1 peptides resulting from tryptic digestion. Squares represent values as fold changes compared to the mean of NAÏVE. Bars correspond to mean +/- SD. Significance was tested with 1-way ANOVA. ns: not significant. Graph summarizes results from 3 independent biological replicates, each biological replicate consists of 3 technical repetitions. (B-C) Western blot of total *C*. *elegans* protein extracted from synchronized adult population, followed by anti-Myc immunoprecipitation, probed with anti-Myc antibody. (B) Detection of Myc-tagged wild-type and RNA-binding mutant MSI-1 protein. (C) Immunoprecipitated Myc-tagged wild-type MSI-1, followed by treatments with or without Lambda phosphatase in absence or presence of phosphatase inhibitor cocktail. Arrows show the two MSI-1 bands with different electrophoretic mobility corresponding to phosphorylated MSI-1 (upper band) and non-phosphorylated MSI-1 (lower band).

Previously, we demonstrated that the *C*. *elegans msi-1* gene actively regulates forgetting of short- and long-term memories [11]. We hypothesized that *C*. *elegans* MSI-1 protein levels could be altered due to changes in gene expression or protein stability upon learning and memory consolidation. To check for possible learning- or memory-related protein changes, total MSI-1 abundance was estimated using mass spectrometry. For the analysis, FLAG-tagged MSI-1 was immunoprecipitated from lysate of worms collected before, right after or 4 hours after conditioning. Using mass spectrometry, we failed to detect a significant learning-induced or memory-related change in total MSI-1 protein levels (Fig 1A). Therefore, MSI-1 activity might be primarily regulated via activity changes mediated by post-translational modifications, rather than by protein expression changes.

### The N-terminal part of MSI-1 is phosphorylated at different residues

Previously, Musashi proteins have been classified in different organisms as phosphoproteins [23–25], therefore, we hypothesized that a possible modulator of MSI-1 activity during forgetting could be phosphorylation. To test this hypothesis, we first explored the phosphorylation status of MSI-1. We analyzed Myc-tagged wild-type MSI-1 protein immunoprecipitated from transgenic *C*. *elegans* extracts expressing the protein under the control of a 3.2 kb long *msi-1* promoter. It was previously shown that the addition of a Myc-tag does not influence MSI-1 function [11]. Using Western blot analysis, we found that *C*. *elegans* MSI-1 migrates as a doublet, indicating that the protein might indeed undergo post-translational modifications (Fig 1B and 1C). To confirm that the two bands correspond to phosphorylated and non-phosphorylated forms of MSI-1, the immunoprecipitated MSI-1 protein was treated with lambda phosphatase prior to loading on an SDS-PAGE and western blot analysis. As expected, the phosphatase treatment resulted in a shift of total MSI-1 to the faster migrating form, which was inhibited by the simultaneous presence of phosphatase and a phosphatase inhibitor cocktail (Fig 1C). Altogether, these results strongly support the hypothesis that MSI-1 is phosphorylated.

### Identification of phosphorylated peptides in the *C*. *elegans* MSI-1 protein

**Fig 2:**
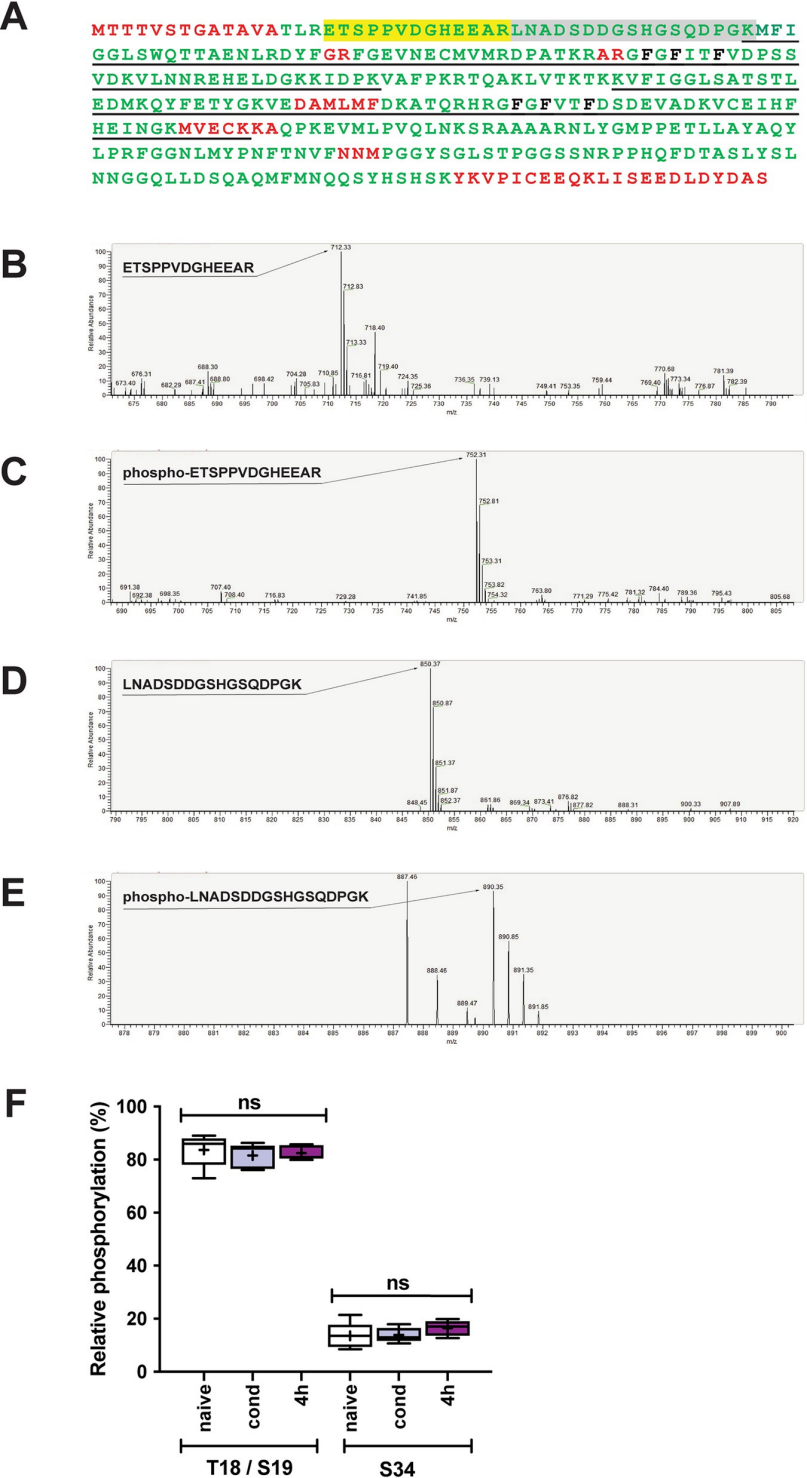
Identification of phosphorylated amino acid residues in *C*. *elegans* MSI-1. (A) Amino acid sequence of the full length MSI-1 protein. Residues covered (green), and missing (red) in the mass spectrometry analysis are depicted. Phosphorylated peptides are highlighted: ETSPPVDGHEEAR (yellow) and LNADSDDGSHGSQDPGK (grey). The two RNA-binding domains are underlined (black) depicting RRM1 and RRM2 respectively. Amino acids highlighted in black show highly conserved residues essential for RNA binding. All of these were mutated in the MSI-1 RNA-binding mutant. (B-E) Mass spectra of the identified phosphorylated peptides and their corresponding non-phosphorylated counterparts. Mass spectra shown are captured at the time point corresponding to the apex of the elution peak (based on liquid chromatography) of the given peptide. The mass difference between phosphorylated and non-phosphorylated isoforms matches the m/z value of phosphate (40 Da). (F) Worms were collected before (naive), right after (cond) or 4 hours after (4H) conditioning, proteins were extracted and MSI-1 was immunoprecipitated. Relative phosphorylation (%) was calculated for each condition (for the details see Materials and methods). Dots represent individual values of relative phosphorylation (%) level of the peptide corresponding to T18/S19 or S34. Bars and whiskers represent mean and SD. Graph summarizes the results from 5 individual biological replicates. Significance was tested using 1-way ANOVA, ns = not significant.

To identify potential phosphorylation sites of MSI-1, we analyzed the post-translational modifications of the protein using LC-MS/MS. Firstly, the *C*. *elegans* MSI-1 protein was reproducibly detected, with an overall high coverage (89%) which was predicted to cover most of the potential phosphorylation sites (Fig 2A). Next, we analyzed the results and detected several post-translational modifications, including phosphorylated ETSPPVDGHEEAR and LNADSDDGSHGSQDPGK peptides. The fragmentation spectrum reproducibly indicated a single phosphorylation event at the threonine or serine residues in ETSPPVDGHEEAR peptide, corresponding to either T18 or S19 respectively (Fig 2C), together with the non-phosphorylated peptide counterpart (Fig 2B). Given the overlapping profiles of the alternatively phosphorylated peptide isoforms, explicit differentiation between T18- or S19-phosphorylated ETSPPVDGHEEAR peptides was not possible. In addition, we found a single phosphorylation of the LNADSDDGSHGSQDPGK fragment at the S34 residue (Fig 2E), along with the non-phosphorylated version of the peptide (Fig 2D). Additionally, mass spectrometry analysis revealed that the ETSPPVDGHEEAR peptide is highly phosphorylated (83.6% ± 6.5), while LNADSDDGSHGSQDPGK peptide phosphorylation is low (13.6% ± 5.0) (Fig 2F) in untrained worms. Finally, we addressed whether there could be learning- or memory-related changes in the phosphorylation state of the detected peptide species. We found that there were no significant detectable changes in the relative phosphorylation of the aforementioned peptides, neither directly after, nor 4 hours after associative learning (Fig 2F). However, our results cannot fully rule out the possibility that cell type-specific or local changes in MSI-1 phosphorylation occur during memory.

### Perturbations of MSI-1 phosphorylation lead to altered memory performance

**Fig 3:**
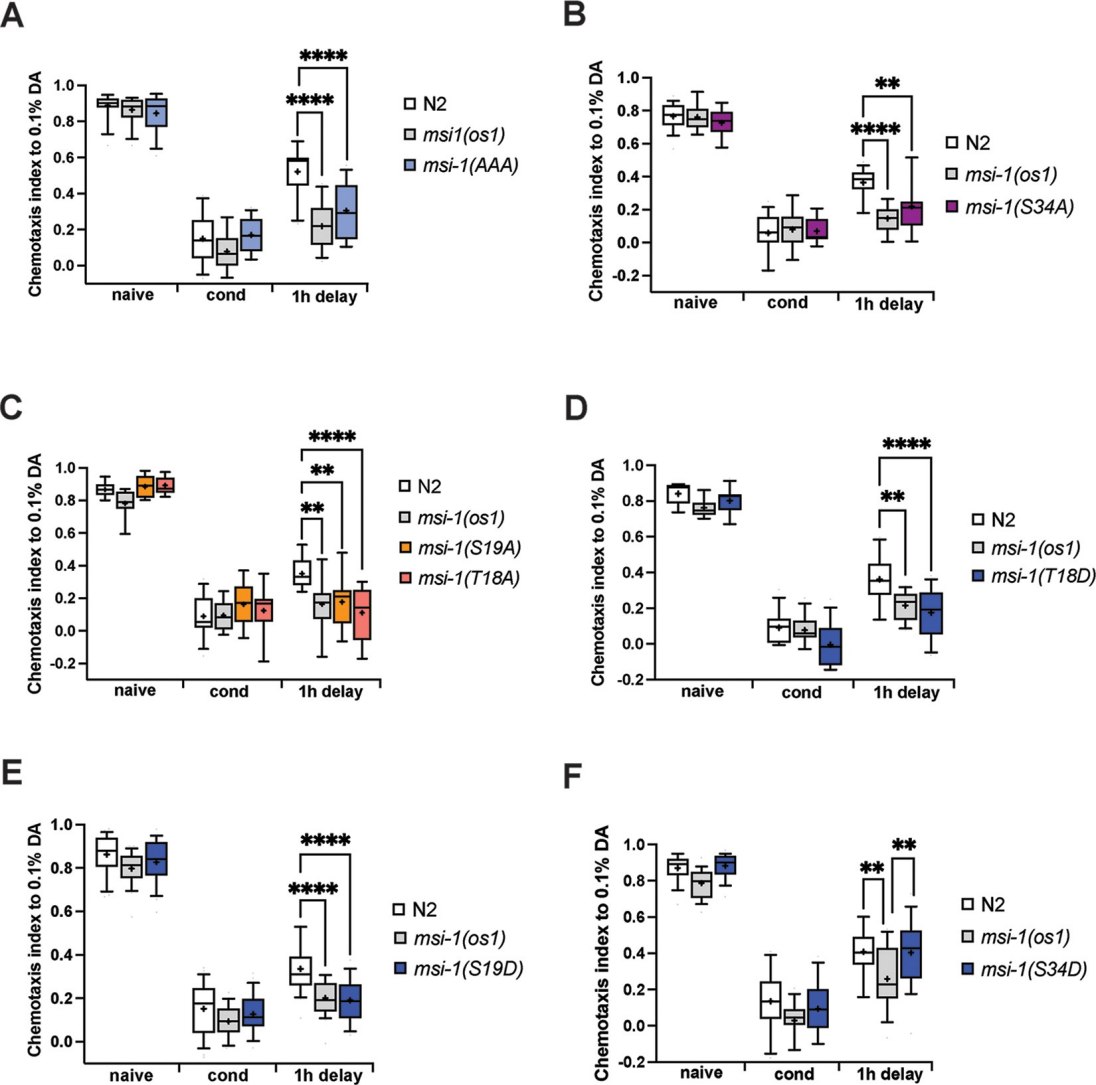
Short-term memory phenotyping of *msi-1* phospho-mutants. Negative olfactory STAM was tested in WT, *msi-1(os1)* and animals with phospho-inhibitory (A-C) or phospho-mimetic (D-F) mutations as indicated. Worms were assayed toward 1:1000 diluted DA before (naive), directly after conditioning (cond) or followed by a 1-hour recovery phase (1h delay). All experiments were done in triplicates and repeated at least four times. Data is represented in boxplots with 10 and 90 percentile whiskers. Significance was tested with 2-way ANOVA and *post hoc t-tests* across all conditions. ns = not significant, asterisks represent Bonferroni-corrected p-values: * = p<0.05, ** = p<0.01 *** = p<0.001 and **** = p<0.0001.

**Fig 4:**
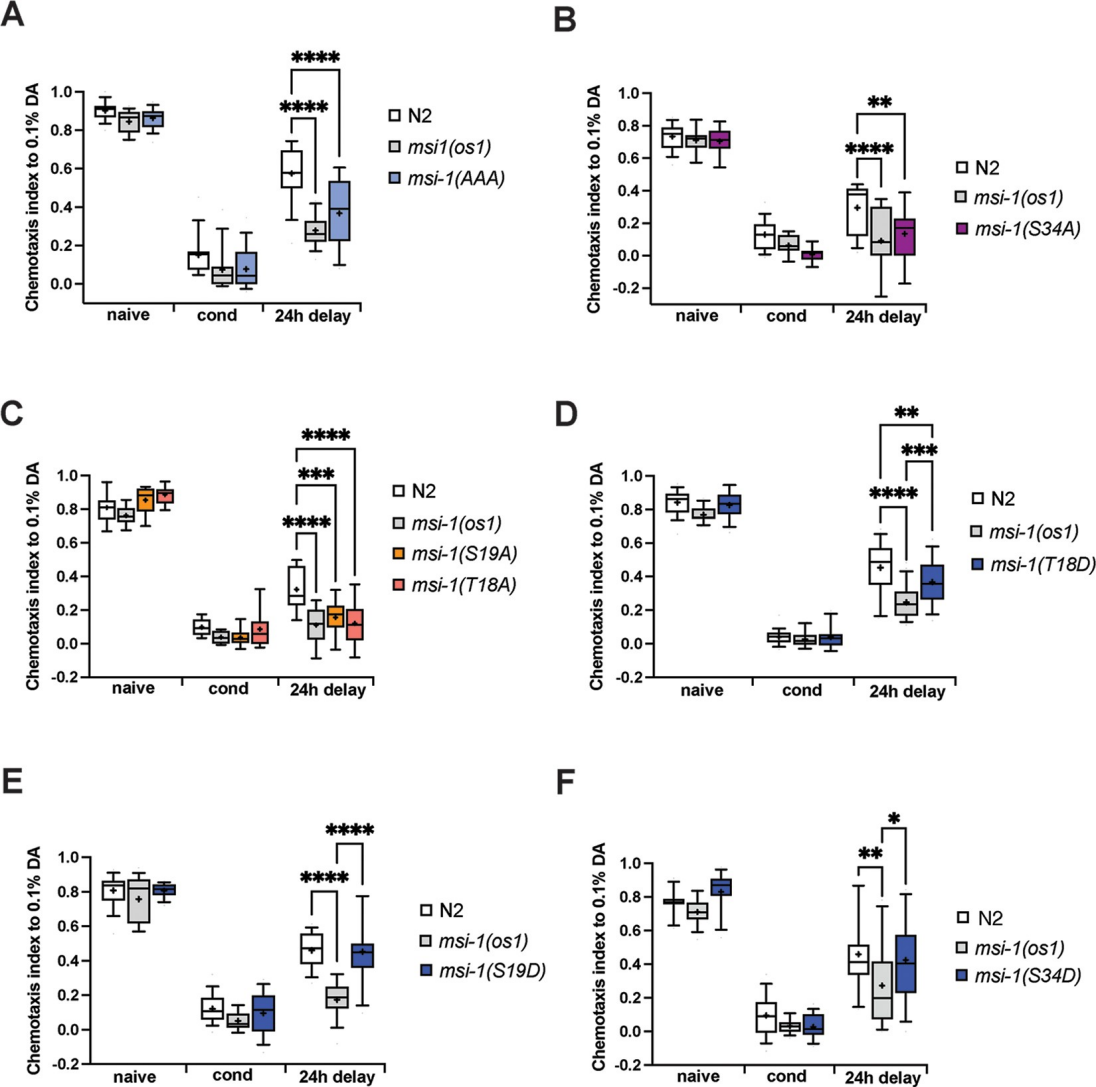
Long-term memory phenotyping of *msi-1* phospho-mutants. Negative olfactory LTAM was tested in WT, *msi-1(os1)* and animals with phospho-inhibitory (A-C) or phospho-mimetic (D-F) mutations as indicated. Worms were assayed toward 1:1000 diluted DA before (naive), directly after conditioning (cond) or followed by a 24-hour recovery phase (24h delay). All experiments were done in triplicates and repeated at least four times. Data is represented in boxplots with 10 and 90 percentile whiskers. Significance was tested with 2-way ANOVA and *post hoc t-tests*. ns = not significant, asterisks represent Bonferroni-corrected p-values: * = p<0.05, ** = p<0.01 *** = p<0.001 and **** = p<0.0001.

To study the importance of MSI-1 phosphorylation during memory in *C*. *elegans*, we introduced various phospho-inhibitory (T/S to A) or phospho-mimetic mutations (T/S to D) in the endogenous protein using CRISPR/Cas9 genome editing [27]. None of these mutations affected baseline chemotaxis (Figs 3 and 4). Furthermore, the 1-hour (STAM) or 2-hour (LTAM) starvation period in the presence of diacetyl (DA) evoked a decreased chemotaxis towards DA; suggesting that a lack of phosphorylation or an emulated constitutive phosphorylation at the sites in question does not interfere with the learning process (Figs 3 and 4). The decreased response of wild-type worms to DA is not due to sensory adaptation, since conditioned wild-type worms retain the ability to detect and respond normally towards 2,4,5-trimethylthiazole, an odour sensed by the AWA neuron (S1A Fig). Additionally, we used *odr-10(ky32)* mutants [28] as a negative control to ensure that starvation alone is not responsible for the behavior of worms. As previously shown [28], we observed that *odr-10* naïve worms do not respond to low concentrations of DA but respond normally to 2,4,5-trimethylthiazole (S1B Fig). After conditioning, *odr-10* worms retain an intact response to 2,4,5-trimethylthiazole, suggesting that starvation alone is not responsible for the conditioned behavior of worms to DA. To investigate the effects of phospho-inhibitory mutations on memory performance, we assayed the various mutant strains and found that in worms carrying either the simultaneous or individual phospho-inhibitory mutations, short-term memory retention was significantly greater compared to wild-type worms and similar to *msi-1(os1)* deletion mutant worms (Fig 3A–3C). Interestingly, we found that phospho-mimetic mutations of T18D/S19D sites showed a similar phenotype to phospho-inhibitory mutations when assessing short-term memory (Fig 3D and 3E), while *msi-1(S34D)* exhibited a wild-type STAM phenotype (Fig 3F).

Similar to short-term memory, the simultaneous introduction of the T18A, S19A and S34A phospho-inhibitory mutations increased long-term memory retention similar to *msi-1(os1)* mutant worms (Fig 4A). Similarly, individual substitutions of either S34A, S19A or T18A significantly increased long-term memory retention (Fig 4B and 4C). Separate mutations of S19 or S34 sites to aspartic acid did not interfere with LTAM performance when compared to WT worms (Fig 4E and 4F), whilst the T18D conversion resulted only in a subtle effect on LTAM compared to wild-type worms (Fig 4D). Together, these results show that the phosphorylation state of MSI-1 plays a crucial role in the memory-related function of the protein in *C*. *elegans* (summarized in Table 1). The effect on LTAM is not due to any injury/arousal (e.g. pseudoconditioning) or some other permanent damage that might occur during the training paradigm, since wild-type worms show a decreased response to DA 24h after training whilst showing intact responsiveness to 2,4,5-trimethylthiazole (S1A Fig). Finally, the LTAM phenotype is not due to any potential effect starvation might exert, as *odr-10* mutants show intact response to 2,4,5-trimethylthiazole 24hrs after conditioning (S1B Fig).

**Table 1:** Summary of short-term (STAM) and long-term (LTAM) memory phenotypes of the various MSI-1 phospho-mutants.

Genotype	STAM phenotype	LTAM phenotype
*os1*	Enhanced memory retention	Enhanced memory retention
*T18A;S19A;S34A*	Enhanced memory retention	Enhanced memory retention
*T18A;S19A*	Enhanced memory retention	Enhanced memory retention
*T18A*	Enhanced memory retention	intermediate
*S19A*	Enhanced memory retention	Enhanced memory retention
*S34A*	Enhanced memory retention	Enhanced memory retention
*T18D*	Enhanced memory retention	Wild-type
*S19D*	Enhanced memory retention	Wild-type
*S34D*	Wild-type	Wild-type

### MSI-1 protein abundance does not change in the AVA interneuron upon introduction of phospho-inhibitory mutations

**Fig 5:**
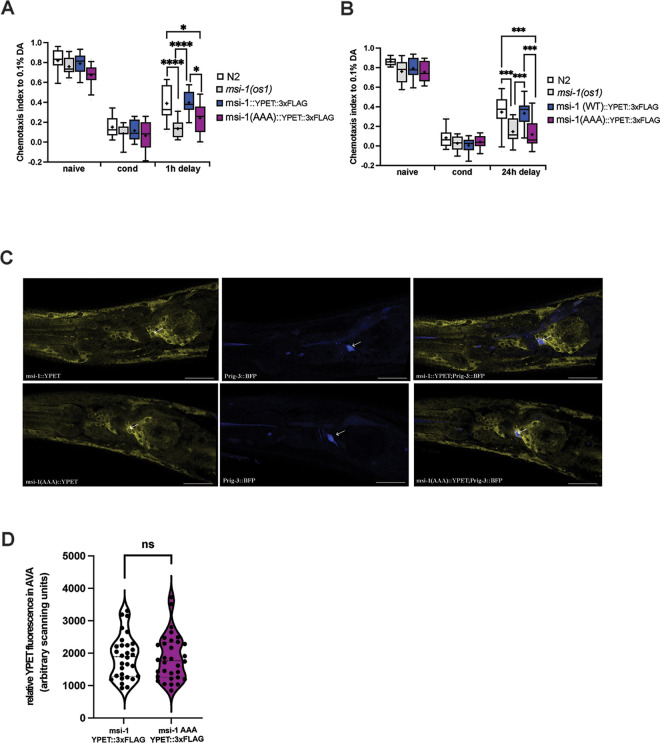
MSI-1 protein abundance in AVA interneuron remains unchanged upon simultaneous introduction of phospho-inhibitory mutations. Negative olfactory (A) STAM and (B) LTAM were tested in WT, *msi-1(os1)*, MSI-1:YPET::3xFLAG and MSI-1 (AAA)::YPET::3xFLAG animals. Worms were assayed toward 1:1000 diluted DA before (naive), directly after conditioning (cond) or followed by a 1 or 24-hour recovery phase (1h, 24h delay). All experiments were done in triplicates and repeated at least four times. Data is represented in boxplots with 10 and 90 percentile whiskers. Significance was tested with 2-way ANOVA and *post hoc t-tests* across all conditions. ns = not significant, asterisks represent Bonferroni-corrected p-values: * = p<0.05, ** = p<0.01 *** = p<0.001 and **** = p<0.0001. (C) Representative confocal images of MSI-1 expression in the AVA interneuron (arrows) in MSI-1::YPET::3xFLAG and MSI-1(AAA)::YPET::3xFLAG animals. Scale bar, 20μm. (D) Violin plots displaying quantification of fluorescence intensity in AVA interneuron in MSI-1::YPET::3xFLAG and MSI-1(AAA)::YPET::3xFLAG animals. For each genotype animals were recorded with identical microscope settings and YPET intensity was measured on z-projected confocal images and quantified using ImageJ software. The centre line of the violin plot represents the group median, whereas the bottom and top lines represent the 25^**th**^ and 75^**th**^ percentiles respectively. Statistical analysis was performed using a two-tailed unpaired Student’s t-test, ns: not significant.

MSI-1 is expressed in multiple tissues of adult worms such as seam, muscle and gut cells as well as neurons [11]. Therefore, we cannot exclude the possibility that changes to the phosphorylation status of the protein could alter MSI-1 expression in neurons. For this reason, we measured MSI-1 protein levels in the AVA interneuron, where MSI-1 was previously shown to play a role in modulating forgetting [11]. We first tagged the MSI-1 wild-type protein as well as the MSI-1 protein harboring all three phospho-inhibitory mutations with YPET and 3xFLAG. We find that tagging the endogenous protein with CRISPR/Cas9 is the most appropriate technique to assess true protein expression changes and circumvents limitations which have been reported when using other methods such as multicopy extrachromosomal arrays or mos1-mediated Single Copy Insertion (mosSCI) [29]. To ensure that the tag does not interfere with protein function, we tested the tagged wild-type MSI-1 carrying strain in an aversive olfactory chemotaxis assay and found no changes in baseline behavior, learning, STAM or LTAM memory performance while in accordance to the previous results the triple T/S to A mutant strain showed a similar phenotype to the *msi-1(lf)* mutant worms (Fig 5A and 5B). Next, we crossed the *msi-1*::*YPET*::*3xFLAG* and *msi-1(AAA)*::*YPET*::*3xFLAG* lines to an integrated *rig-3* promoter-driven BFP line, in order to easily and reliably identify AVA (Fig 5C). The simultaneous introduction of the T18A, S19A and S34A phospho-inhibitory mutations did not affect MSI-1 abundance when compared to wild-type worms (Fig 5D); suggesting that phosphorylation does not influence protein levels but rather regulates protein activity.

## Discussion

In a previous study we reported that loss of *msi-1* in *C*. *elegans* leads to enhanced memory retention [11], however, the underlying regulatory mechanisms controlling MSI-1 activity were unknown. Here, we investigated changes of MSI-1 protein abundance and effect of post-translational modifications on the activity of MSI-1 during memory.

First, we tested the MSI-1 protein levels and could not detect any changes in total MSI-1 protein abundance during learning and memory (Fig 1A), suggesting that MSI-1 activity might be modulated by post-translational modifications rather than at the protein level. However, we cannot fully rule out subtle or tissue-specific protein level changes other than in AVA neuron that could happen during memory formation.

In accordance with the possible role of post-translational modifications, we showed that *C*. *elegans* MSI-1 protein is phosphorylated at residues T18, S19 and S34 at the N-terminal end of the protein and that this phosphorylation plays a crucial role in the protein’s function in associative memory (Figs 2–4). Furthermore, we have shown that RNA-binding could affect the MSI-1 phosphorylation status, since the phosphorylation of the RNA-binding mutant was impaired (Fig 1B). Thus, our results suggest that the interaction of MSI-1 with its target RNAs is necessary for MSI-1 phosphorylation and that RNA might play an important role for the assembly and generation of an active MSI-1 protein complex. In line with this hypothesis, links between RNA-binding and protein phosphorylation have been established [30–32]. This suggests that phosphorylation may, indeed, affect the interaction of MSI-1 with its downstream RNA targets; such as ARX-1, ARX-2 and/or ARX-3, subunits of the Arp2/3 actin branching regulator complex, previously identified to interact with MSI-1 [11].

Interestingly, interference of MSI-1 phosphorylation through individual or simultaneous T/S to A mutations coherently phenocopied the *msi-1* loss-of-function phenotype for both short- and long-term memory (Figs 3A–3C and 4A–4C). Thus, our results show that phosphorylation at these threonine and serine residues is essential for MSI-1 activity. The effects of the phospho-inhibitory mutations are not additive, since the memory phenotype of the simultaneous T/S to A mutations is comparable to that of the single mutations (Figs 3A–3C and 4A–4C).

Based on our mass spectrometry analysis it was not possible to discriminate whether T18 or S19 is the phosphorylated residue in ETSPPVDGHEEAR trypsin-digested MSI-1 peptide (Fig 2C). Our memory phenotyping assays indicate that phosphorylation of either S19 or T18 is important for MSI-1 activity. Consistent with this hypothesis, *msi-1(T18A)* show a similar STAM as well as LTAM phenotype to *msi-1(S19A)* mutants. However, the substitution of T18 with aspartic acid does not fully emulate phosphorylation (Fig 4D), which might be due to the fact that T18 modifications abrogate *msi-1* function in long-term memory irrespective of phosphorylation. In addition, we observed substantial differences in short- versus long-term memory performance of *msi-1(T18D)* and *msi-1(S19D)* mutant worms (Figs 3D, 3E and 4D, 4E), which could be attributed to distinct underlying molecular characteristics, raising the possibility that phosphorylation-dependent MSI-1 promoted forgetting does not equally contribute to the erasure of short- and long-term memories. This is in line with previous reports suggesting a distinct nature of the molecular mechanisms underlying short- and long-term memory processing [33,34].

Strikingly, we found that both T/S to A or T/S to D substitutions of T18 and S19 sites confer enhanced short-term memory retention (Fig 3C–3E). The observed identical STAM phenotypes of phospho-inhibitory and phospho-mimetic mutations might be explained by the fact that aspartic acid fails to fully mimic phospho-serine or phospho-threonine in their negative charge, size, or geometry. Consequently, aspartic acid substitutions may not be able to entirely recapitulate the effects of phosphorylation, which results in a loss-of-function phenotype as it has been reported elsewhere [35].

In our study we detected a second (S34) phosphorylation site which showed an overall lower relative phosphorylation compared to the T18/S19 site (Fig 2D and 2F). The S34A substitution in MSI-1 protein consistently results in both short- and long-term enhanced memory retention (Figs 3B and 4B), indicating the essential role of phosphorylation at this site regardless of the paradigm used (short-term or long-term). Furthermore, *msi-1(S34D)* displayed a wild-type phenotype for both short- and long-term memory (Figs 3F and 4F). Mass spectrometry data showed no changes in relative phosphorylation of any of the peptides identified upon learning and memory (Fig 2F), indicating that S34 site is most likely constantly phosphorylated. Taken together phosphorylation at S34 likely plays a permissive instead of a regulatory role. However, we cannot exclude that there might be tissue-specific changes occurring, for example in the AVA interneuron, which might be masked from the signal coming from other tissues.

Finally, we showed that changes in the phosphorylation status of MSI-1 do not affect protein levels in AVA neuron (Fig 5D). Consequently, phosphorylation is likely to cause changes in protein activity, ultimately affecting MSI-1 binding affinity to its downstream targets during aversive olfactory associative memory.

Altogether, our study highlights the critical role of post-translational modifications of MSI-1 in *C*. *elegans* and demonstrates that phosphorylation is essential for the activity of MSI-1 during forgetting.

## Materials and methods

### General methods and *C*. *elegans* strains used

Common reagents were obtained from Sigma (Sigma-Aldrich, St Louis, MO) unless otherwise indicated. Standard methods were used for maintaining and manipulating *C*. *elegans* [36]. The *C*. *elegans* Bristol strain, variety N2, was used as wild-type reference in all experiments. To purify plasmids for microinjection we used midiprep plasmid purification kit (Qiagen, Hilden, Germany). Extrachromosomal array expressing transgenic lines were generated by injecting DNA at a concentration of 10–100 ng/μl into both arms of the syncytial gonad of young adult worms as described previously [37,38]. p_*sur-5*_::*mDsRed* or p_*myo-2*_::*mCherry* were used as transformation markers at 10 and 2.5 ng/μl concentration respectively. Chromosomal integration of extrachromosomal arrays was done by UV irradiation [37]. Genome editing of the target gene loci was performed using co-CRISPR/Cas9 strategy as described previously [27]. All generated strains were four times backcrossed to the wild-type strain. The *C*. *elegans* alleles and strains used in this study were: *msi-1(os1)*, *msi-1(os1); utrIs3[p*_*msi-1*_::*msi-1cDNA*::*MYC-tag*::*3’UTR*, *p*_*sur-5*_::*mDsRed]*, *msi-1(os1); utrIs17[p*_*msi-1*_::*msi-1cDNA*::*3xFLAG*::*3’UTR*, *p*_*myo-2*_::*mCherry]*, *msi-1(utr12[T18A*,*S19A*,*S34A])*, *msi-1(utr17[T18A])*, *msi-1(utr18[S19A])*, *msi-1(utr7[S34A])*, *msi-1(utr46[T18D])*, *msi-1(utr43[S19D])*, *msi-1(utr16[S34D])*, *msi-1(utr55[YPET*::*3xFLAG])*, *msi-1(utr64[YPET*::*3xFLAG*::*msi-1T18A*,*S19A*,*S34A])*, *msi-1(utr55[YPET*::*3xFLAG]); utrSi43[p*_*rig-3*_::*LoxP*::*BFP*::*LoxP*::*FLP-D5*::*SL2*::*GFP*::*H2B]*, *unc-119(ed3)*, *msi-1(utr64[YPET*::*3xFLAG*::*msi-1S18A*,*T19A*,*S34A]); utrSi43[p*_*rig-3*_::*LoxP*::*BFP*::*LoxP*::*FLP-D5*::*SL2*::*GFP*::*H2B]*,*unc-119(ed3)*, *odr-10 (ky32)*.

### Genome editing with CRISPR-Cas9

Modification of the endogenous loci was performed using Co-CRISPR/Cas9 genome editing method described previously [39]. sgRNA was designed to direct Cas9 cleavage at the desired locus using ApE plasmid editor software based on the previously published sequence requirements [27]. For Co-CRISPR genome editing, sgRNA targeting the locus of interest, sgRNA targeting the *dpy-10* locus, repair oligonucleotides (Microsynth AG, Balgach, Switzerland) for *dpy-10* and for *msi-1* with the desired modifications and a plasmid allowing expression of Cas9 in the germline were co-injected into the gonad of young adult worms. F1 animals showing roller phenotype were singled and allowed to propagate. To confirm the presence of CRISPR-Cas9-initiated modifications, worms were genotyped with PCR using the following primers: 5’-CAGCAGAAGCAGCAGCATCAG-3’ and 5’-TGTGAGAAGTAAAAACGGAGCAAAC-3’. The primers amplify a 500 base pair long DNA from the *msi-1* locus. Amplified PCR products were subjected to digestion with SalI enzyme (New England Biolabs, Ipswich, MA) resulting in two smaller fragments if the CRISPR/Cas9-modified T18/S19 alleles were present. Similarly, the amplified PCR products were digested with HpyCH4V or MseI enzymes (New England Biolabs, Ipswich, MA) to detect the presence of *msi-(S34A)* or *msi-1(S34D)* alleles, respectively. The genotype of the animals was confirmed with sequencing (Microsynth AG, Balgach, Switzerland). Homozygous animals carrying the modified allele were backcrossed four times.

Endogenous tagging of *msi-1* with YPET::3xFLAG was generated as previously described [40]. Briefly, 601bp and 722bp homology arms flanking the N-terminus of *msi-1* were PCR amplified from N2 genomic DNA and inserted into the mNG^SEC&3xFlag vector pDD283 using NEBuilder Hifi DNA assembly (New England Biolabs, Ipswich, MA). The Cas9 target site was selected using the Sequence Scan for CRISPR database (http://cistrome.org/SSC/) and inserted into pDD162 [41]. The sgRNA sequence used was 5’- ATGACAACGACAGTATCAACGTTTTAGAGCTAGAAATAGCAAGT-3’. A mixture of 50 ng/μl Cas9–sgRNA plasmid, 10 ng/μl repair template, and 2.5 ng/μl pCFJ90, 5 ng/μl pCFJ104 and 10 ng/μl *sur-5p*::*mdsRed* co-injection markers was injected into the gonads of young adults (Mello et al., 1991). Knock-in line was established, SEC cassette was excised using heat shock and the *msi-1* YPET::3xFLAG line was sequenced to verify correct insertion of the tag.

### Chemotaxis assays

Chemotaxis to diacetyl was investigated in synchronized one-day-old young adult populations as previously described [42]. Briefly, worms were washed three times with CTX solution (5 mM KH_2_PO_4_/K_2_HPO_4_ pH 6.0, 1 mM CaCl_2_, and 1 mM MgSO_4_) and approximately 80–150 worms were placed in the middle of a 10 cm CTX test plate (1.9% agar, 5 mM KH_2_PO_4_/K_2_HPO_4_ pH 6.0, 1 mM CaCl_2_, and 1 mM MgSO_4_). Worms were given a choice between a spot of either diacetyl (1:1000) or 2,4,5-trimethylthiazole (1:1000) diluted in ethanol versus a control spot of ethanol. Additionally, 1μl of 20mM sodium azide was used to paralyze the worms that reached the chosen spot. The distribution of the worms on the testing plate was determined after 1 hour and the chemotaxis index (number of worms in the diacetyl spot minus number of worms in the ethanol spot divided by the total number of worms on the plate) was calculated as described previously [42]. Short-term or long-term aversive olfactory conditioning was performed as previously described [11,43]. Briefly, for the short-term assays, worms were subjected to starvation for 1 hour in the presence of 2μl diacetyl on 10 cm CTX plates. Following training chemotaxis of the worms towards diacetyl was assessed directly or after a 1-hour resting period on CTX plates without bacteria. Long-term memory consolidation was induced with 2 times repeated 1-hour training session with a 30 minutes rest in presence of food in between. Following conditioning, worms were kept on NGM plates seeded with OP_50_ bacteria for 24 hours and tested for diacetyl chemotaxis after the recovery phase.

### Protein extraction and immunoprecipitation

For immunoprecipitation, synchronized one-day old-adult worms (grown for 96 hours at 20 degrees after egg lay) were collected in ice-cold RIPA buffer (50mM Tris-HCl pH 7.5, 150mM NaCl, 1% Triton-X-100, 0.5% sodium-deoxycholate, 0.1% SDS, 1mM EDTA, 10 mM NaF, 1 mM Na-orthovanadate) supplemented with protease inhibitor cocktail (Roche, Basel, Switzerland). Samples were homogenized in Mixer Mill MM 301 (Retsch GmbH, Germany) for 30s repeated four times. Lysates were cleaned by centrifugation at 13.000 rpm for 20 min at 4°C. Protein concentration of the supernatant was measured using Pierce BCA Protein Assay Kit (ThermoFisher Scientific, Waltham, MA) according to the manufacturer’s instructions. Anti-FLAG M2 Affinity Gel (Sigma Aldrich, St. Louis, MI) and Anti-c-Myc Agarose Affinity Gel (Sigma Aldrich, St. Louis, MI) bead-conjugated antibodies were used to overnight immunoprecipitate the protein of interest from 2 mg total worm protein extract. Following incubation, samples were washed 3 times with HNTG buffer (50mM HEPES pH 7.4, 150mM NaCl, 10% glycerol, 1% Triton-X-100).

### Western blot analysis

Samples were subjected to SDS-PAGE, transferred to PVDF membranes, blocked with 5% non-fat dry milk in TBST (50mM Tris-HCl, pH 7.5, 150mM NaCl, 0.05% Tween-20) and incubated with primary antibodies as indicated. Antibodies used were: mouse anti-c-Myc 9E10 (1:1000, Thermo Fisher Scientific, Waltham, MA), mouse anti-FLAG (1:1000, Sigma Aldrich, St. Louis, MI) and mouse anti-actin (1:2000, Merck Millipore, Burlington, MA). Primary antibodies were detected using HRP coupled secondary antibodies (1:5000, Jackson ImmunoResearch Laboratories, Cambridge House, UK). Chemiluminescent signal was developed using Clarity and ClarityMax Western Blotting Substrates (BioRad Laboratories Inc., Hercules, CA) followed by detection with a FujiFilm ImageQuant LAS-4000 detector (GE Healthcare, Chicago, IL).

### Mass spectrometry analysis

For the identification of post-translational modifications of *C*. *elegans* MSI-1, liquid chromatography with tandem mass spectrometry (LC-MS/MS) was used. Immunoprecipitated protein samples were subjected to reduction (20mM dithiothreitol, 50mM Tris-HCl pH 8.0) and alkylation (50mM iodoacetamide, 50mM Tris-HCl, pH 8.0) followed by overnight tryptic, chymotryptic, AspN and Lys-C (Promega, Madison, WI) digestions. Next, the digested samples were acidified with TFA (1% in water), then desalted using Vydac C18 Silica Microspin columns (5–200μl, 5–60μg, The Nest Group Inc., Southborough, MA), followed by elution with 80% acetonitrile/0.1% TFA solution. Samples were separated using reverse phase liquid chromatography (New Objectives, MA) and the eluted peptides were ionized and analyzed with Orbitrap FT hybrid mass spectrometer attached to LTQ instrument (Thermo Fisher Scientific, Waltham, MA). Resulting MS/MS spectra were evaluated with Proteome Discoverer 1.4 (Thermo Fisher Scientific, Waltham, MA) using Mascot (Matrix Science, London, UK) as search engine. Mass spectra were annotated using consensus and processing workflows, phosphorylated amino acid residues were identified and the relative abundance of peptides was estimated using Proteome Discoverer 1.4 software (Thermo Fisher Scientific, Waltham, MA). Relative phosphorylation of peptides was calculated as follows:

$$Relative\;phosphorylation\;(\%)=\frac{Abundance\;(phosphorylated)}{Abundance\;(phosphorylated)+Abundance\;(non-phosphorylated)}\;X\;100$$

### Fluorescence microscopy

Whole worms were mounted on 3% agar pads and immobilized with 0.5% sodium azide (NaN_**3**_). Synchronized one-day old adult worms (grown for 96 hours at 20 degrees after egg lay) were imaged using a Zeiss LSM 880 scanning confocal microscope equipped with a 63x oil immersion objective. Images were processed and quantified using ImageJ. For quantification of the fluorescence intensity in AVA, the integrated intensity value was calculated for each image and its corresponding background subtracted. Due to the dense neuronal signal in the head of the animal, AVA was first localized and selected in the BFP channel and the identical selection was then superimposed onto the YPET channel.

### Statistical analysis

All data was analyzed using Prism 9 software (GraphPad Software Inc., San Diego, CA). Main effects and interaction terms were investigated using ANOVA. The p-value threshold was set to nominal significance (p < 0.05). Pairwise group comparison was tested using *post hoc* t-tests corrected for multiple comparisons using Bonferroni correction (pBonf. < 0.05). For the imaging quantification, a two-tailed unpaired Student’s t-test was carried out to assess any differences between groups and the p-value threshold was set to nominal significance (p < 0.05). All raw data used in the study can be found in S1 Table.

## Supporting information

S1 Fig Aversive olfactory associative learning and LTAM are not due to sensory neuron adaptation or starvation. (A) Negative olfactory learning and LTAM towards DA were tested in WT animals. Baseline chemotaxis of worms was initially assayed toward 1:1000 diluted DA and 1:1000 diluted 2,4,5-trimethylthiazole (naive). Worms were conditioned in the presence of DA and absence of food for two rounds of 1h each and tested directly after conditioning (cond) or followed by 24-hour recovery phase (24h delay) toward 1:1000 diluted DA and 1:1000 diluted 2,4,5-trimethylthiazole. (B) Negative olfactory learning and LTAM towards DA were tested in WT and *odr-10* animals. Baseline chemotaxis of worms was initially assayed toward 1:1000 diluted DA and 1:1000 diluted 2,4,5-trimethylthiazole (naive). Worms were conditioned in the presence of DA and absence of food for two rounds of 1h each and tested directly after conditioning (cond) or followed by 24-hour recovery phase (24h delay) toward 1:1000 diluted DA and 1:1000 diluted 2,4,5-trimethylthiazole. All experiments were done in triplicates and repeated at least six times. Data is represented in boxplots with 10 and 90 percentile whiskers. Significance was tested with 2-way ANOVA and *post hoc t-tests* across all conditions. ns = not significant, asterisks represent Bonferroni-corrected p-values: *** = p<0.001 and **** = p<0.0001.
(TIF)

S1 Table Raw data for Figs 1–5. Raw numerical values used for plots presented on Figs 1–5.
(XLSX)
