# Supplementary figures and images for: Phosphorylation of MSI-1 is implicated in the regulation of associative memory in Caenorhabditis elegans
Source: PLoS Genet. 2022 Oct 12;18(10):e1010420. doi: 10.1371/journal.pgen.1010420 (PMC9555661; doi:10.1371/journal.pgen.1010420)

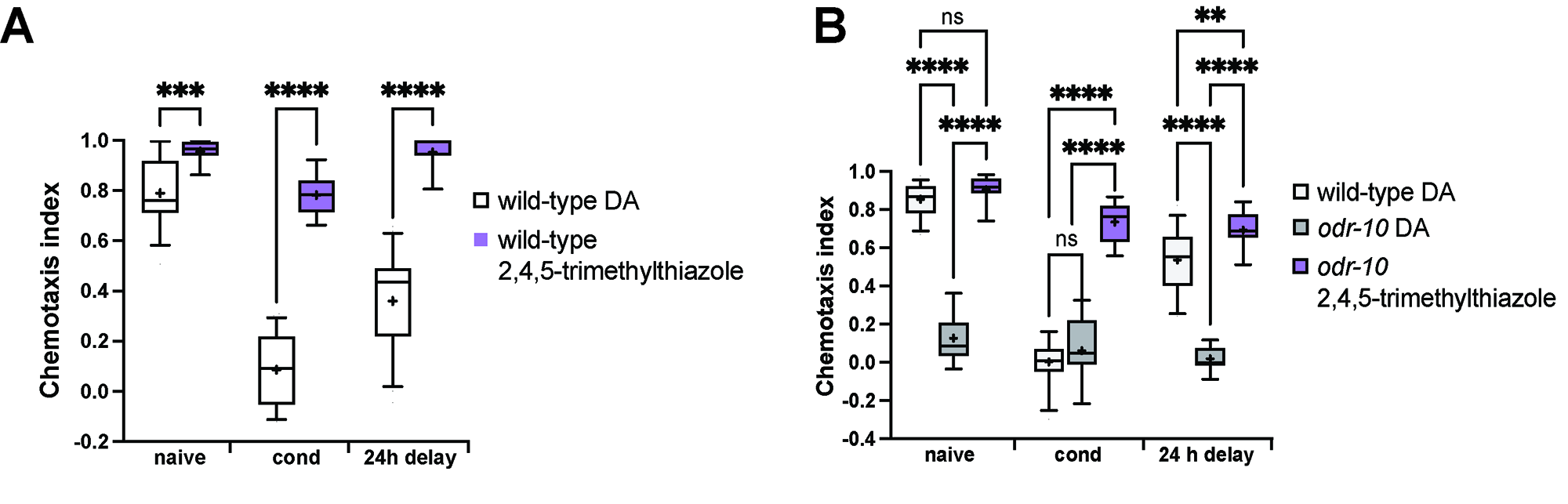

Supplement: S1 Fig — (A) Negative olfactory learning and LTAM towards DA were tested in WT animals. Baseline chemotaxis of worms was initially assayed toward 1:1000 diluted DA and 1:1000 diluted 2,4,5-trimethylthiazole (naive). Worms were conditioned in the presence of DA and absence of food for two rounds of 1h each and tested directly after conditioning (cond) or followed by 24-hour recovery phase (24h delay) toward 1:1000 diluted DA and 1:1000 diluted 2,4,5-trimethylthiazole. (B) Negative olfactory learning and LTAM towards DA were tested in WT and odr-10 animals. Baseline chemotaxis of worms was initially assayed toward 1:1000 diluted DA and 1:1000 diluted 2,4,5-trimethylthiazole (naive). Worms were conditioned in the presence of DA and absence of food for two rounds of 1h each and tested directly after conditioning (cond) or followed by 24-hour recovery phase (24h delay) toward 1:1000 diluted DA and 1:1000 diluted 2,4,5-trimethylthiazole. All experiments were done in triplicates and repeated at least six times. Data is represented in boxplots with 10 and 90 percentile whiskers. Significance was tested with 2-way ANOVA and post hoc t-tests across all conditions. ns = not significant, asterisks represent Bonferroni-corrected p-values: *** = p<0.001 and **** = p<0.0001. (TIF) [file pgen.1010420.s001.tif]
